# Serum Metabolomics Reveals Serotonin as a Predictor of Severe Dengue in the Early Phase of Dengue Fever

**DOI:** 10.1371/journal.pntd.0004607

**Published:** 2016-04-07

**Authors:** Liang Cui, Yie Hou Lee, Tun Linn Thein, Jinling Fang, Junxiong Pang, Eng Eong Ooi, Yee Sin Leo, Choon Nam Ong, Steven R. Tannenbaum

**Affiliations:** 1 Infectious Diseases Interdisciplinary Research Group, Singapore-MIT Alliance for Research and Technology (SMART), Singapore; 2 KK Research Centre, KK Women’s and Children’s Hospital, Singapore; 3 Communicable Disease Center, Institute of Infectious Diseases and Epidemiology, Tan Tock Seng Hospital, Singapore; 4 Saw Swee Hock School of Public Health, National University of Singapore, Singapore; 5 Emerging Infectious Diseases Program, Duke-NUS Graduate Medical School, Singapore; 6 NUS Environment Research Institute, National University of Singapore, Singapore; 7 Departments of Biological Engineering and Chemistry, Massachusetts Institute of Technology, Cambridge, Massachusetts, United States of America; University of Rhode Island, UNITED STATES

## Abstract

Effective triage of dengue patients early in the disease course for in- or out-patient management would be useful for optimal healthcare resource utilization while minimizing poor clinical outcome due to delayed intervention. Yet, early prognosis of severe dengue is hampered by the heterogeneity in clinical presentation and routine hematological and biochemical measurements in dengue patients that collectively correlates poorly with eventual clinical outcome. Herein, untargeted liquid-chromatography mass spectrometry metabolomics of serum from patients with dengue fever (DF) and dengue hemorrhagic fever (DHF) in the febrile phase (<96 h) was used to globally probe the serum metabolome to uncover early prognostic biomarkers of DHF. We identified 20 metabolites that are differentially enriched (*p*<0.05, fold change >1.5) in the serum, among which are two products of tryptophan metabolism–serotonin and kynurenine. Serotonin, involved in platelet aggregation and activation decreased significantly, whereas kynurenine, an immunomodulator, increased significantly in patients with DHF, consistent with thrombocytopenia and immunopathology in severe dengue. To sensitively and accurately evaluate serotonin levels as prognostic biomarkers, we implemented stable-isotope dilution mass spectrometry and used convalescence samples as their own controls. DHF serotonin was significantly 1.98 fold lower in febrile compared to convalescence phase, and significantly 1.76 fold lower compared to DF in the febrile phase of illness. Thus, serotonin alone provided good prognostic utility (Area Under Curve, AUC of serotonin = 0.8). Additionally, immune mediators associated with DHF may further increase the predictive ability than just serotonin alone. Nine cytokines, including IFN-γ, IL-1β, IL-4, IL-8, G-CSF, MIP-1β, FGF basic, TNFα and RANTES were significantly different between DF and DHF, among which IFN-γ ranked top by multivariate statistics. Combining serotonin and IFN-γ improved the prognosis performance (AUC = 0.92, sensitivity = 77.8%, specificity = 95.8%), suggesting this duplex panel as accurate metrics for the early prognosis of DHF.

## Author Summary

Dengue, an acute arboviral disease has emerged globally, inflicting debilitating symptoms in 96 million people. The early prediction of severe dengue (dengue hemorrhagic fever, DHF) is challenging due to varied and late-presenting symptoms, requiring frequent monitoring for signs of disease progression. An often used parameter to monitor disease progression is decrease in platelet count, or thrombocytopenia, which is a feature of DHF. However, whether platelet-derived compounds are useful as early biomarkers predictive of DHF has not been investigated. In this study, we investigated the utility of serum metabolites as predictive biomarkers. We developed quantitative and high-throughput tools and discovered circulating serotonin, conceivably platelet-derived, that showed a nearly two-fold decrease in DHF patients compared to mild dengue fever. Because immune mediators may increase the predictive ability, we measured them in blood and identified interferon-gamma as an important cytokine in DHF. When serotonin is used in combination with IFN-γ, this dual-panel predictive panel provides accurate prognosis of DHF within 96 h from fever onset. These findings may have important clinical implications not just in early dengue prognostication but also in the design of therapeutic strategies against dengue infections.

## Introduction

Dengue is arguably the most important arboviral disease globally, with an estimated 390 million infections occurring yearly, of which nearly 100 million are clinically apparent [1]. The clinical manifestations of dengue infections range from mild undifferentiated febrile illness to classical dengue fever (DF), severe dengue hemorrhagic fever (DHF) and sometimes fatal disease. The two predominant pathophysiologic observations in DHF are increased vascular permeability and thrombocytopenia, which result in plasma leakage and increased risk of hemorrhage, respectively. Early triage in the acute, febrile phase is useful for streamlining case management and monitoring.

One of the pathophysiological hallmarks of severe dengue is increased vascular permeability, leading to a loss in blood volume that if uncorrected, could lead to shock. The degree of plasma leakage varies and prediction of severe plasma leakage has been challenging [2]. Daily or even more frequent monitoring of hematocrit levels may be required for at least 2–3 days around the period of fever defervescence to detect plasma leakage soon after its onset. As timely fluid support prevents progression of plasma leakage to hypovolemic shock and multi-organ failure, tools that enable triaging of patients according to disease outcome could enable a more effective use of limited healthcare resources, especially during dengue epidemics [3]. Indeed, the components of early pathophysiological mechanisms could serve as reliable prognostic factors given their mechanistic role in disease manifestation.

It is generally believed that the observed pathophysiology is immune-mediated brought about by monocytes, T-cells, endothelial cells, mast cells and increasingly platelets, as well as the interactions between these cell types [4–7]. Despite the consistent presence of thrombocytopenia in the acute stages of dengue, little research has focused on the pathologic contribution of platelet-derived soluble factors in DHF progression and their use as prognostic markers. Platelets circulate in high numbers throughout the system and upon activation release their granule contents to exert their hemostatic, immunological and inflammatory effects [8,9]. DENV has been reported to activate platelets [6]. One of these bioactive compounds released during platelet activation is serotonin (5-hydroxytryptamine or 5-HT). Platelets store serotonin in the dense granules but lack the enzymes to synthesize serotonin. Instead, platelets take up plasma serotonin produced by enterochromaffin cells in the gastrointestinal tract, through the serotonin receptor (SERT or 5-HTT) [10]. Serotonin is secreted during platelet activation and promotes platelet aggregation. It also further amplifies platelet activation and aggregation through 5-HT_2A_ receptor re-uptake to result in vasoconstriction of surrounding blood vessels and hemostasis [11]. While *in vitro* results suggest DENV induced platelet apoptosis [6], the reduction of platelet number and function has not been characterized *in vivo*. Furthermore, thrombocytopenia and associated platelet dysfunction is short-lived and the rapid recovery of platelet numbers in the convalescence phase suggest that the function of soluble metabolites and/or immune mediators with short half lives may play critical roles in disease progression.

In this study, we adopted a metabolomics to study dengue-induced metabolites, especially platelet-derived molecules and evaluated their potential as prognostic biomarkers for severe dengue. We discovered a major reduction in circulating serotonin in both DF and DHF, with the reduction in DHF exceeding that of DF. In addition, serotonin levels correlated with the degree of thrombocytopenia, and when used in combination with IFN-γ, they provide accurate early (<96 h from onset of fever) prognosis of DHF.

## Methods

### Patient enrollment

The dengue study cohort of 116 dengue patients– 60 DF patients and 56 DHF patients (Table 1) were recruited from the Prospective Adult Dengue Study (PADS) [12]. Briefly, PADS is a cohort study of acutely febrile adults at a tertiary care center, Communicable Diseases Center, Tan Tock Seng Hospital, Singapore. Adult patients (≥ 18 years) presenting with acute onset of fever (≥ 37.5°C) without rhinitis or other clinical alternatives were included in the study (Febrile stage, < 96 hours post onset of fever; Defervescence, Day 5–7, Convalescence, Day 21–28). Venous blood samples were collected, aliquoted and frozen at -80°C for hematological, virological and serological analysis. Enrollment of all eligible individuals was based on written informed consent and the collected samples were anonymized. The protocols were approved by the Domain Specific Review Board of the National Healthcare Group, Singapore (DSRB/E/2009/432). We reported our study design, hypotheses, patient characteristics, assay methods, statistical methods and modeling methods as per REMARK which is important for generalizability [13]. Additionally we used serum samples from 24 asymptomatic age- and gender-matched healthy subjects as controls. DF and DHF patients were classified according to the WHO 1997 dengue guidelines [14]. To fulfill the case definition of DHF, all four of the following criteria must be present, namely: fever or history of fever, hemorrhagic tendencies, thrombocytopenia and evidence of plasma leakage [14]. Hematoconcentration was determined by the hematological analyzer and expressed as % of the volume of whole blood that was made up of red blood cells. Hematocrit increase of over 20% of the values at convalescence phase is considered a common clinical index of plasma leakage and DHF diagnosis. The PADS cohort comprised of both DF and DHF patients recruited at different phases of dengue infection–(febrile phase: DF = 25; DHF = 27; defervescence phase: DF = 31; DHF = 29, convalescence phase: DF = 25; DHF = 25). DENV2 is the predominant DENV type. Based on the anti-DENV IgG and IgM seropositivity or seronegativitiy, immune status of the patients was determined. Anti-DENV IgM seropositivity and IgG seronegativity indicated that 40–52% of DF and 22–41% of DHF are primary cases, and the remaining secondary cases. 12–16% DF patients and 59–76% DHF patients had platelets <50×10^3^/μL at any point as determined during their daily routine total blood count.

**Table 1:** Patient characteristics and demographics.

Phase	Characteristic	DF	DHF
**Febrile**	*N*	25	27
** **	Mean age (range), *y*	33.9 (21–44)	40.8 (22–67)
	Average fever days	3.5	3.6
** **	Primary infection (%)	10 (40)	6 (22)
** **	Secondary infection (%)	15 (60)	21 (78)
** **	Serotype 1	3	2
** **	Serotype 2	22	22
** **	Serotype 3	0	1
** **	Serotype 4	0	2
** **	<50k Platelets (%)	0 (0%)	3 (11%)
	Mean platelet count (x10^3/μL)	118.0	87.0
**Defervescence**	*N*	31	29
** **	Mean age (range), *y*	34.9 (25–56)	37.7 (20–64)
	Average fever days	5.4	5.6
** **	Primary infection (%)	16 (52)	12 (41)
** **	Secondary infection (%)	15 (48)	17 (59)
** **	Serotype 1	5	3
** **	Serotype 2	24	22
** **	Serotype 3	2	3
** **	Serotype 4	0	1
** **	<50k Platelets (%)	3 (10)	6 (21)
	Mean platelet count (x10^3/μL)	101.5	75.9
**Convalescence**	*N*	25	25
** **	Mean age (range), *y*	35.9 (21–49)	37.4 (20–59)
	Average fever days	26.2	26.0
** **	Primary infection (%)	10 (40)	7 (28)
** **	Secondary infection (%)	15 (60)	18 (72)
** **	Serotype 1	2	3
** **	Serotype 2	23	17
	Serotype 3	0	2
	Serotype 4	0	3
	<50k Platelets (%)	0 (0)	0 (0)
	Mean platelet count (x10^3/μL)	218.2	218.2

### Hematological, serological and virological analysis

A detailed hematological and virological analysis was performed and included white blood cell count (WBC), red blood cell count (RBC), blood hemoglobin (HGB), hematocrit (HCT), mean corpuscular volume (MCV), mean corpuscular hemoglobin (MCH), mean corpuscular hemoglobin concentration (MCHC), platelet count (PLT), lymphocyte percentage (LYMPH%), lymphocyte count (LYMPH), mixed cell count (MXD), neutrophil percentage (NEUT%), neutrophil count (NEUT), red blood cell distribution width-coefficient of variation (RDW-CV), and quantitation of peripheral viral titers using reverse transcriptase-polymerase chain reaction (RT-PCR) crossover values (C_t_). Dengue viral infection was confirmed by RT-PCR [15], or NS1 detection by Dengue NS1 Ag Strip (Bio-Rad, Marnes-la-Coquette, France) at the Environmental Health Institute, Singapore, or typing by virus isolation and immunofluorescence using DENV type-specific monoclonal antibodies (ATCC: HB46-49). Dengue-immune status (primary or secondary DENV infection) was based on Dengue IgG levels in the acute sera, using a commercially obtained ELISA (PanBio, Brisbane, Australia) according to the manufacturer’s protocol.

### Sample preparation

For untargeted metabolomics analysis, a volume of 50 μL from each serum sample was thawed at 4°C and serum proteins were precipitated with 200 mL ice-cold methanol, which contained 10 mg/mL 9-fluorenylmethoxycarbonyl-glycine as an internal standard. After vortexing, the mixture was centrifuged at 16,000 rpm for 10 minutes at 4°C and the supernatant was collected and evaporated to dryness in a vacuum evaporator. The dry extracts were then redissolved in 200 μL of 98:2 water/methanol for liquid chromatography-mass spectrometry (LC-MS) analysis. Quality control (QC) samples were prepared by mixing equal amounts of serum samples from all the samples and processed as per other samples. The QC sample was run after each 8 samples to monitor the stability of the system and all samples were randomized.

For targeted metabolomics analysis, sample preparation followed a published report with some modifications [16]. Briefly, 10 μL of the internal standard mix was added to 50 μL of serum. The sample was then diluted to 100 μL with water containing 0.1% formic acid (v/v), vortexed, followed by the addition of 400 μL of cold methanol. After vortexing, the mixture was centrifuged at 16,000 rpm for 10 minutes at 4°C and the supernatant was collected and evaporated to dryness in a vacuum evaporator. The dry extracts were then redissolved in 200 μL of 0.1% formic acid in water for LC-MS/MS analysis. A summary of the workflow utilized in untargeted and targeted metabolomics studies is shown in S1 Fig.

### Relative quantification by untargeted metabolomics

Untargeted metabolomics were performed as previously described with modifications [17]. The supernatant fraction from sample preparation step was analyzed using Agilent 1290 ultrahigh pressure liquid chromatography system (Waldbronn, Germany) equipped with a 6520 QTOF mass detector managed by a MassHunter workstation. The column used for the separation was an Agilent rapid resolution HT Zorbax SB-C18 (2.1×100 mm, 1.8 mm; Agilent Technologies, Santa Clara, CA, USA). The oven temperature was set at 45°C. The gradient elution involved a mobile phase consisting of (A) 0.1% formic acid in water and (B) 0.1% formic acid in methanol. The initial condition was set at 5% B. A 7 min linear gradient to 70% B was applied, followed by a 12 min gradient to 100% B which was held for 3 min, then returned to starting conditions over 0.1 min. Flow rate was set at 0.4 ml/min, and 5 mL of samples was injected. The electrospray ionization mass spectra were acquired in both positive and negative ion mode. Mass data were collected between m/z 100 and 1000 at a rate of two scans per second. The ion spray voltage was set at 4,000 V, and the heated capillary temperature was maintained at 350°C. The drying gas and nebulizer nitrogen gas flow rates were 12.0 L/min and 50 psi, respectively. Two reference masses were continuously infused to the system to allow constant mass correction during the run: m/z 121.0509 (C_5_H_4_N_4_) and m/z 922.0098 (C_18_H_18_O_6_N_3_P_3_F_24_).

### Absolute quantification by targeted metabolomics

The targeted LC-MS/MS analysis followed a published report with some modifications [16]. Briefly, LC-MS analysis was performed with Agilent 1290 ultrahigh pressure liquid chromatography system (Waldbronn, Germany) coupled to an electrospray ionization with iFunnel Technology on a triple quadrupole mass spectrometer. Chromatographic separation was achieved by using Atlantis T3 column (2.1×100 mm, 3 μm; Waters, Eschbornn, Germany) with mobile phases (A) 0.1% formic acid in water and (B) 0.1% formic acid in methanol. The initial condition was set at 0% B. A 10 min linear gradient to 40% B was applied, followed by 1 min gradient to 100% B which was held for 5 min, then returned to starting conditions over 0.1 min. The column was kept at 40°C and the flow rate was 0.4 mL/min. The auto-sampler was cooled at 4°C and an injection volume of 5 μL was used. Electrospray ionization was performed in positive ion mode with the following source parameters: drying gas temperature 200°C with a flow of 14 L/min, nebulizer gas pressure 30 psi, sheath gas temperature 400°C with a flow of 11 L/min, capillary voltage 3,000 V and nozzle voltage 800 V. Compounds were quantified in multiple reaction monitoring (MRM) mode with the following transitions: *m/z* 177>160, *m/z* 181>164, *m/z* 209>192, *m/z* 213>196, *m/z* 233>174, *m/z* 192>146, and *m/z* 213>196 for serotonin, d_4_-serotonin, kynurenine, and d_4_-kynurenine, melatonin, 5-hydroxy-indole-3-acetic acid (HIAA), and 5-hydroxytryptophan (HTP), respectively. Data acquisition and processing were performed using MassHunter software (Agilent Technologies, US). A representative LC-MS/MS chromatogram of a native standards mix is shown in S2 Fig.

The method was validated for limit of detection (LOD), linearity, accuracy, precision and recovery, according to Food and Drug Administration (FDA) guidelines for biological method as previous published report [16]. Briefly, the calibration curves were constructed from three replicate measurements of eight concentrations of each standard. A linear regression with r^2^ >0.995 was obtained in all relevant ranges. The LODs, defined by a signal-to-noise ratio (S/N) of 3, ranged from 0.5 to 10.0 nM for all the analytes. The recoveries were evaluated by spiking defined amounts of analytes into aliquots of unprocessed serum and analyte concentrations were calculated using the calibration curves. The spiked concentration was obtained by subtracting the endogenous concentration which was determined from the analysis of the unspiked sample. The recoveries generally ranged from 52.4% to 82.6%. For intra-batch and inter-batch precision and accuracy, the relative standard deviation (RSD) values ranged from 1.1% to 13.2% all the analytes.

### Cytokine analysis

Fluorescent bead based measurement of cytokines, chemokines and growth factors in the patients’ sera were performed in duplicates using the Luminex technology xMAP (Bioplex 27-plex human cytokine kit, Bio-Rad, California, USA) as per manufacturer’s instructions. The measured analytes are IL-1β, IL-1ra, IL-2, IL-4, IL-5, IL-6, IL-7, IL-8, IL-9, IL-10, IL-12, IL-13, IL-15, IL-17, basic FGF, Eotaxin, G-CSF, GM-CSF, IFN-γ, IP-10, MCP-1 (MCAF), MIP-1α, MIP-1β, PDGF-BB, RANTES, TNF-α and VEGF. The standard curves were optimized automatically by the software (Bioplex manager) and verified manually. In order to prevent batch effect, samples were randomized prior to analysis. Calibrations and validations were performed prior to analyses.

### Data analysis

Raw spectrometric data in untargeted metabolomics were analyzed by MassHunter Qualitative Analysis software (Agilent Technologies, US) and the molecular features characterized by retention time (RT), chromatographic peak intensity and accurate mass, were obtained by using the Molecular Feature Extractor algorithm. The features were then analyzed by MassHunter Mass Profiler Professional software (Agilent Technologies, US). Only features with an intensity ≥ 20,000 counts (approximately three times the limit of detection of our LC-MS instrument), and found in at least 80% of the samples at the same sampling time point signal were kept for further processing. Next, a tolerance window of 0.15 min and 2 mDa was used for alignment of RT and *m/z* values, and the data normalized to spiked 9-fluorenylmethoxycarbonyl-glycine internal standard. Raw spectrometric data in targeted metabolomics were processed using MassHunter Workstation Quantitative Analysis software (Agilent Technologies, US).

For statistical analysis, nonparametric Test (Wilcoxon, Mann–Whitney test) with Benjamini-Hochberg Multiple Testing Correction was employed, because the samples analyzed were obtained from different patients, and statistical significance was set at *p*<0.05. For multivariate data analysis using hierarchical clustering or Orthogonal projections to latent structures discriminant analysis (OPLS-DA), data were normalized by median-centering and dividing by standard deviation. Hierarchical clustering was performed using MeV version 4.9.0. OPLS-DA was performed using the software package SIMCA-P 13.0 version (Umetrics AB, Umea, Sweden). Metabolites and cytokines/chemokines with Variable Importance in the Projection (VIP) values>1 were considered to be influential for the separation of samples in OPLS-DA analysis. In addition, the fold change (FC) analysis was also performed to further filter the features and only those features with FC > 1.5 were selected as potential significantly altered metabolites. Receiver operating characteristic (ROC) curve was made by using R package.

### Compound identification

The structure identification of the differential metabolites was based on our published work [17]. Briefly, the elemental compositions of the metabolites were first calculated based on the exact mass, the nitrogen rule and the isotope pattern by Masshunter software from Agilent. Then, the elemental composition and exact mass were used for open source database searching, including LIPIDMAPS (http://www.lipidmaps.org/), HMDB (http://www.hmdb.ca/), METLIN (http://metlin.scripps.edu/) and MassBank (http://www.massbank.jp/). Next, MS/MS experiments were performed to obtain structural information via the interpretation of the fragmentation pattern of the metabolite. The MS/MS spectra of possible metabolite candidates in the databases were also searched and compared. Finally, the metabolites were confirmed by comparison with the standards where commercially available, which was the case for serotonin and kynurenine. The metabolites are listed according to the minimum reporting standards for chemical analysis in metabolomics recommended by Metabolomics Standard Initiative (MSI) [18,19]. Briefly, a four-level system ranging from Level 1 (identified metabolites) via Levels 2 and 3 (putatively annotated compounds and compound classes) to Level 4 (unidentified or unclassified metabolites which can still be differentiated based on spectrum data).

## Results

### Global-scale metabolomics revealed early changes in serum metabolome associating with platelet numbers in DHF patients

**Fig 1:**
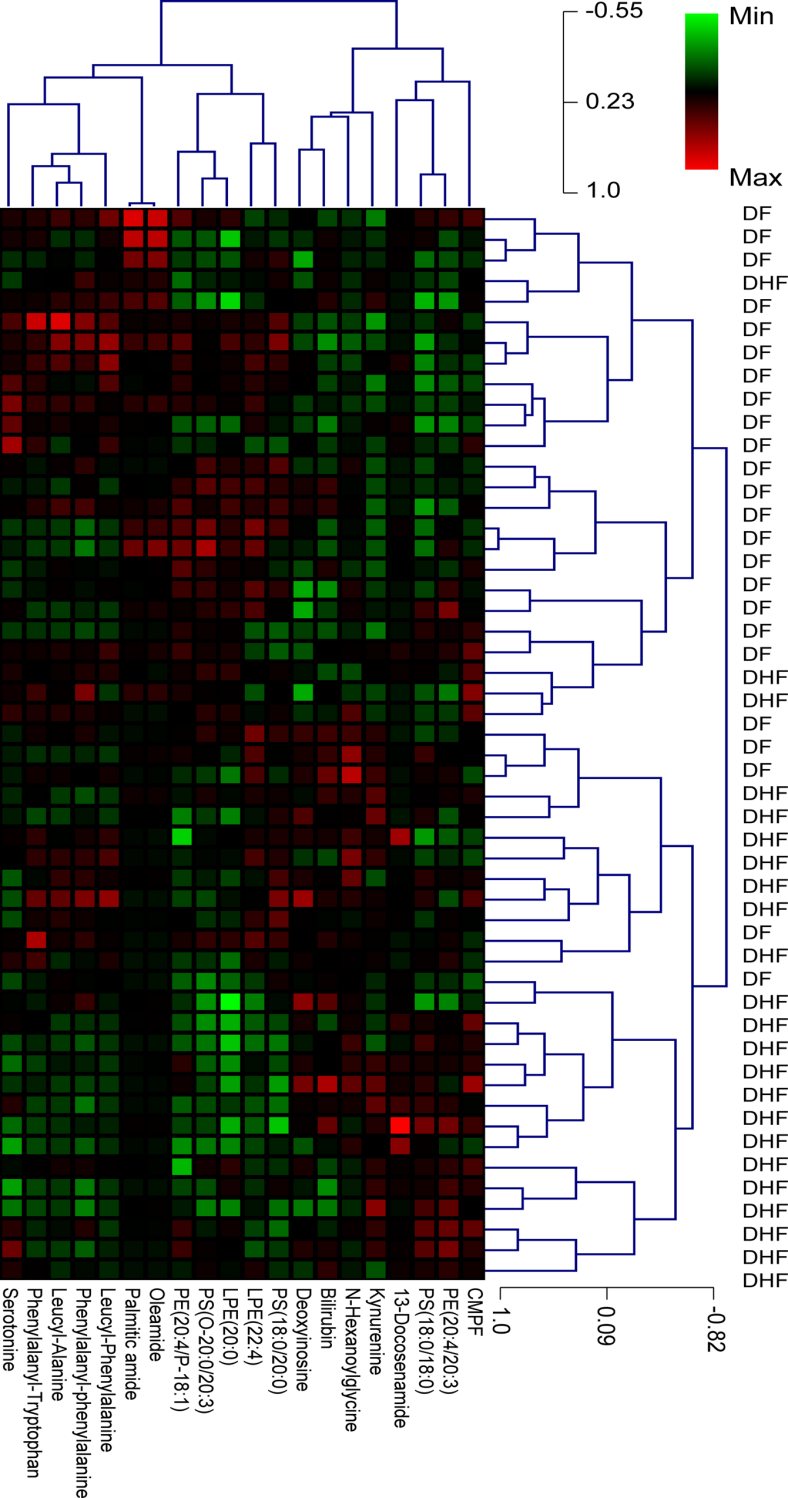
Hierarchical heatmap clustering with identified differential metabolites segregates febrile phase dengue fever (DF) and dengue hemorrhagic fever (DHF) patients. Each column shows ion intensity for a specific metabolite after mean centering and unit variance scaling of the data. Each row shows the serum metabolic profiles of DF and DHF patients.

**Fig 2:**
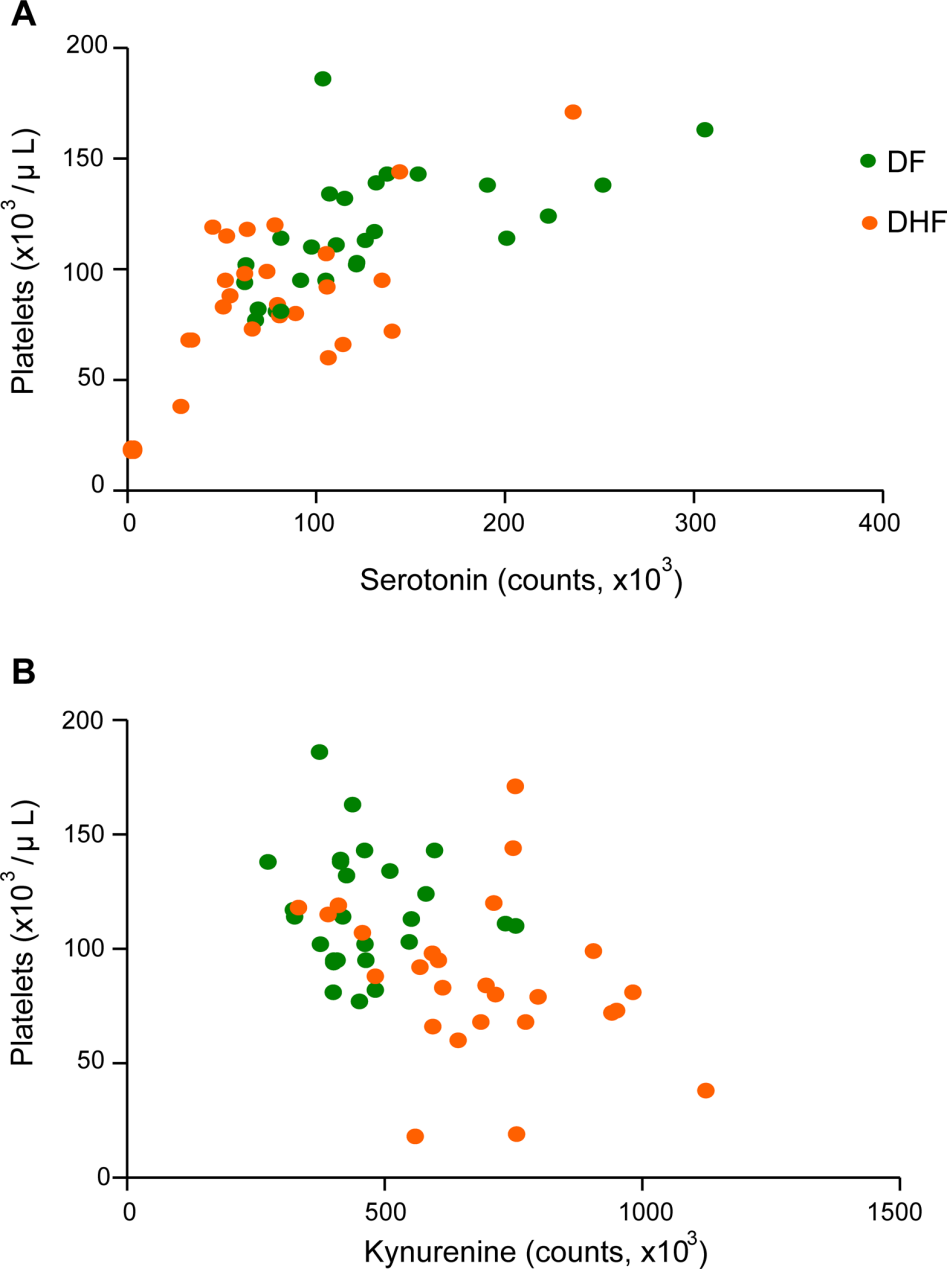
Pearson correlation analysis reveals correlation of platelets with serotonin and kynurenine in the febrile phase of infection. (A) Positive correlation between platelet and serotonin (*r* = 0.67; *p*<0.0001). (B) Negative correlation between platelet and kynurenine (*r* = ‒0.45; *p*<0.005). DF—dengue fever; DHF—dengue hemorrhagic fever.

**Fig 3:**
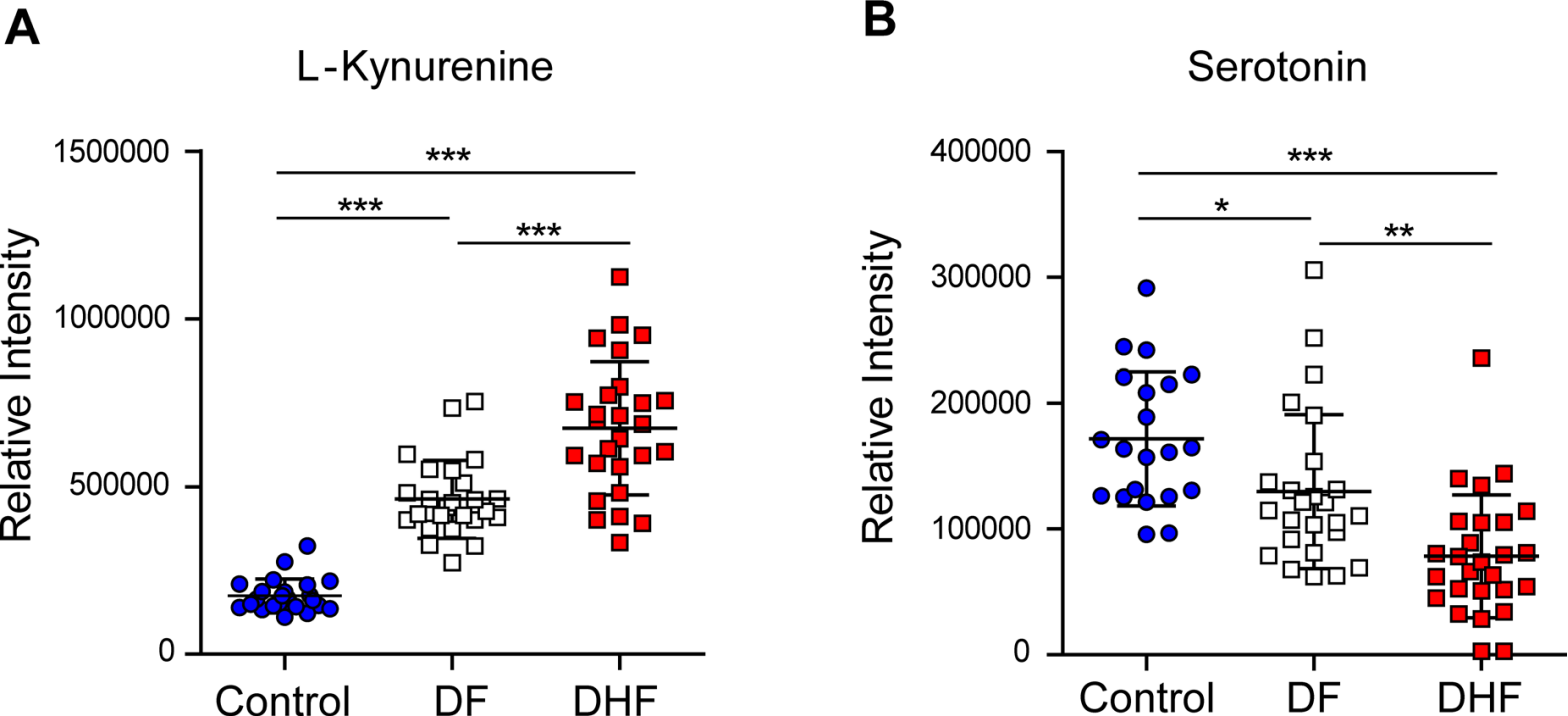
Serum serotonin and kynurenine changes in dengue patients in the febrile phase of infection. (A) Elevated change trend of kynurenine from Control, dengue fever (DF) to dengue hemorrhagic fever (DHF). (B) Decreased change trend of serotonin from Control, dengue fever (DF) to dengue hemorrhagic fever (DHF). Mean value (middle lines) ± standard deviation (SD, error bars) is reported. Significance is indicated as *,*p*<0.05; **,*p*<0.01; ***,*p*< 0.001 by Mann-Whitney test.

We characterized the metabolome changes early in dengue infections using liquid-chromatography tandem mass spectrometry (LC-MS/MS) to globally map the serum metabolomes from DF (*n* = 25) and DHF (*n* = 27) patients. In this untargeted, global metabolomics, a total of 20 MSI Levels 1 and 2 metabolites were significantly different between DHF and DF patients in the febrile phase, of which 8 were increased [(L-kynurenine, 13E-docosenamide, deoxyinosine, N-Heptanoylglycine, 3-carboxy-4-methyl-5-propyl-2-furanpropionic acid, bilirubin, phosphotidylethanolamine (20:4/20:3), phosphatidylserine (18:0/18:0) and 12 were decreased (Leucyl-phenylalanine, phenylalanyl-tryptophan, leucyl-alanine, palmitic amide, serotonin, oleamide, phenylalanylphenylalanine, lysophosphotidylethanolamine (20:0/0:0), lysophosphotidylethanolamine (22:4/0:0), phosphatidylserine (18:0/20:0), phosphotidylethanolamine (20:4/P-18:1), phosphotidylethanolamine (18:1/22:5)] in DHF patients relative to DF patients (Table 2). MSI Levels 3 and 4 metabolites were listed in S1 Table. Hierarchical clustering based on the metabolome profile optimally segregated the patients (Fig 1), where all but four DF patients and three DHF patients were classified separately, therefore correlating well with WHO 1997 dengue classification scheme. Verification of peaks’ retention time and MS/MS spectrum through the use of synthetic standards provided highly confident identification of serotonin and kynurenine. Among the hematological parameters that are routinely tested in the clinics, platelets correlated most frequently with the metabolites, in particular, demonstrating significant positive correlations to serotonin (*r* = 0.67; *p*<0.0001) and negative correlations to kynurenine (*r* = ‒0.45; *p*<0.005) (Fig 2). Decrease in serotonin levels was accompanied with an increase in kynurenine levels in the febrile phase (Fig 3A and 3B). An increase in indoleamine 2,3-dioxygenase in DF patients was reported previously [20], and is presumably the source of kynurenine.

**Table 2:** Identified differential metabolites between DF and DHF patients in the febrile phase of infection.

HMDB	Accurate mass	Metabolite	Chemical formula	*p* value	Fold change (DHF/DF)	Pathway
HMDB00684	208.0817	L-Kynurenine	C_10_H_12_N_2_O_3_	0.001	1.7	Tryptophan metabolism
HMDB29006	351.1524	phenylalanyl-tryptophan	C_20_H_21_N_3_O_3_	0.007	0.57	Dipeptide
HMDB02117	281.2677	oleamide	C_18_H_35_NO	0.002	0.23	Fatty acid amide
HMDB13243	278.1582	Leucyl-phenylalanine	C_15_H_22_N_2_O_3_	0.02	0.39	Dipeptide
HMDB00583	337.3271	Docosenamide	C_22_H_45_NO	0.02	3.49	Fatty acid amide
HMDB00071	252.0814	Deoxyinosine	C_10_H_12_N_4_O_4_	0.005	1.7	Purine metabolism
HMDB28922	202.1268	Leucyl-Alanine	C_9_H_18_N_2_O_3_	0.01	0.43	Dipeptide
HMDB00259	176.0928	Serotonin	C_10_H_12_N_2_O	0.004	0.64	Tryptophan metabolism
HMDB12273	255.2517	Palmitic amide	C_16_H_33_NO	0.002	0.16	Fatty acid amide
HMDB12378	791.558	PS (18:0/18:0)	C_42_H_82_NO_10_P	0.0001	1.62	Glycerophospholipid
HMDB13302	312.1486	Phenylalanylphenylalanine	C_18_H_20_N_2_O_3_	0.02	0.61	Dipeptide
HMDB00054	584.2643	Bilirubin	C_33_H_36_N_4_O_6_	0.02	1.58	Porphyrin and chlorophyll metabolism
HMDB61112	240.1017	CMPF	C_12_H_16_O_5_	0.03	1.59	Fatty acid
HMDB11523	529.3201	LPE(22:4/0:0)	C_27_H_48_NO_7_P	0.02	0.66	Glycerophospholipid
HMDB09044	791.5411	PE(18:1/22:5)	C45H78NO8P	0.02	0.57	Glycerophospholipid
HMDB09397	789.5349	PE (20:4/20:3)	C_45_H_76_NO_8_P	0.009	1.54	Glycerophospholipid
HMDB09414	749.5394	PE(20:4/P-18:1)	C_43_H_76_NO_7_P	0.007	0.69	Glycerophospholipid
HMDB11511	509.3548	LPE(20:0/0:0)	C_25_H_52_NO_7_P	0.01	0.72	Glycerophospholipid
HMDB10164	819.6028	PS(18:0/20:0)	C_44_H_86_NO_10_P	0.02	0.71	Glycerophospholipid
HMDB13010	187.1177	N-Heptanoylglycine	C_9_H_17_NO_3_	0.009	2.07	Acylglycine

### Determination of Serotonin and Kynurenine levels across the time-course of dengue infection

**Fig 4:**
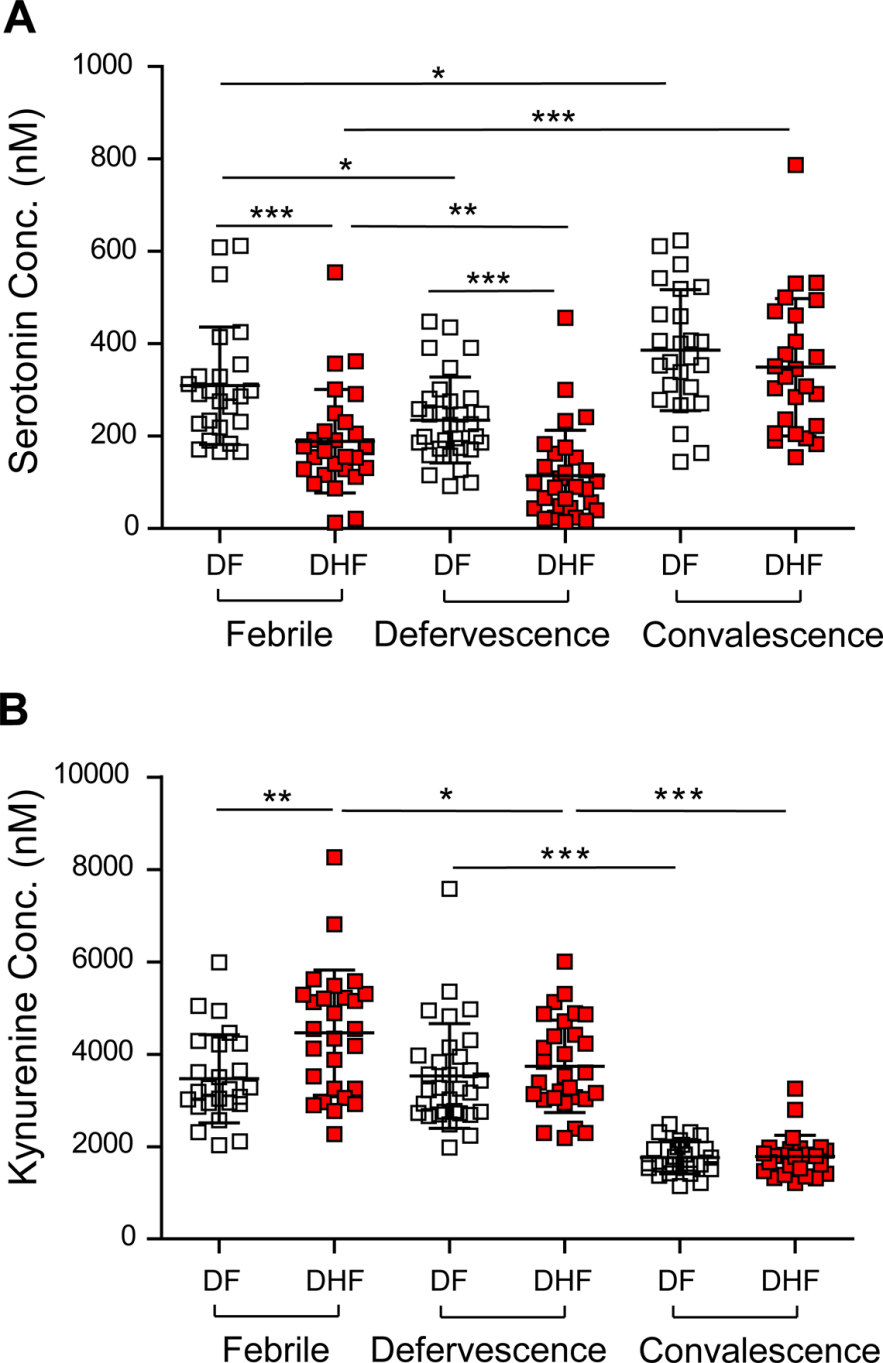
Temporal profile of circulating serotonin and kynurenine in the three main phases of dengue. Temporal profiles of (A) serotonin and (B) kynurenine across the febrile (1–4 d), defervescence (5–7 d) and convalescence (21–28 d) phases. Mean value (middle lines) ± standard deviation (SD, error bars) is reported. Significance is indicated as *, *p*<0.05; **, *p*<0.01; ***, *p*<0.001 by Mann Whitney test. DF—dengue fever; DHF—dengue hemorrhagic fever.

We further evaluated serotonin and kynurenine levels in order to better understand their levels across the course of dengue infection. To improve analytical specificity for absolute quantitative determinations, we developed a high-throughput precision assay based on stable-isotope dilution mass spectrometry of serotonin and kynurenine. Using defined concentrations of deuterated internal standards spiked into samples and calculating the response ratio of the analyte of interest to the internal standard, we accurately determined the concentrations of serotonin and kynurenine in DF and DHF patients (Fig 4A and 4B). Table 3 summarizes the mean values of serotonin and kynurenine (nM) at the three phases in DF and DHF patients. Compared to DF patients, we observed significant decreases in serotonin in DHF patients in both the febrile (*p*<0.001) and the defervescence phases (*p*<0.001), but not the convalescence phase. Kynurenine on the other hand was significantly different between DF and DHF patients in the febrile phase (*p*<0.01), but not in the defervescence and convalescence phases. Furthermore, a dynamic change of serotonin levels was observed in both DF and DHF patients in all three phases of infection. Compared to febrile stage, the level of serotonin continued to decrease and reached the lowest level at defervescence phase. At convalescence phase, increased serotonin was observed and the level was significantly higher than the levels in both febrile and defervescence phases. Meanwhile, there was no difference in serotonin levels between cases of primary and secondary infection in all three phases (S3 Fig).

**Table 3:** Serum serotonin and kynurenine concentrations in DF and DHF patients.

Phase*	Serotonin (mean ± SD, nM)		Kynurenine (mean ± SD, nM)	
	DF	DHF	DF	DHF
**Febrile**	309 ± 127	175 ± 88	3470± 953	4518± 1336
**Defervescence**	231 ± 99	117 ± 102	3597 ± 1166	3613± 993
**Convalescence**	386 ± 131	349. ± 148	1764± 354	1789± 456

* Febrile, DF = 25, DHF = 27; Defervescence, DF = 31, DHF = 29; Convalescence, DF = 25, DHF = 25

Similar to the correlation results in global-scale metabolomics (Fig 2), significant positive correlation between serotonin concentrations and platelet numbers (*r* = 0.55, *p* = 0.0070 (DF) and *r* = 0.67, *p* = 0.0001 (DHF); S4A Fig) was observed in the febrile phase, suggesting that the decrease in serotonin is associated with decreased platelet numbers in DF and DHF patients. Interestingly, the correlation between serotonin and platelets ceased in the defervescence phase (*r* = 0.20, *p*>0.05; S4B Fig). If serotonin levels and platelet counts were organized according to different fever days of dengue patients, serotonin levels continued to decrease from onset of fever up to Day 6 and 7 (S4C Fig), which did not parallel the initial decrease then recovery of platelet numbers with time (S4D Fig).

The levels of HIAA and melatonin, two main degradation products of serotonin, and HTP, the precursor of serotonin, were also evaluated, by using d_4_-serotonin as their internal standard. While the levels of melatonin in serum were below the detection limit, the concentrations of HTP and HIAA in DF and DHF patients were determined (S5A and S5B Fig). Unlike serotonin, neither HTP nor HIAA showed any significant difference between DF and DHF in both the febrile and the defervescence phases.

### Serotonin and IFN-γ as early prognosis biomarkers of DHF

Given the early significant changes of serotonin and kynurenine in DHF patients, and the numerous roles of serotonin in platelet aggregation and activation [11] and kynurenine in immunomodulation [21], Receiver Operating Curve analyses were performed to assess their prognostic utility. The higher performing AUC of serotonin (AUC = 0.80, 95% C.I. 0.68–0.92, *p* = 0.0002) shows its prognostic superiority compared to kynurenine (AUC = 0.72, 95% C.I. 0.57–0.86, *p* = 0.008) (S6A and S6B Fig), and was henceforth selected as a better prognostic biomarker for DHF.

**Fig 5:**
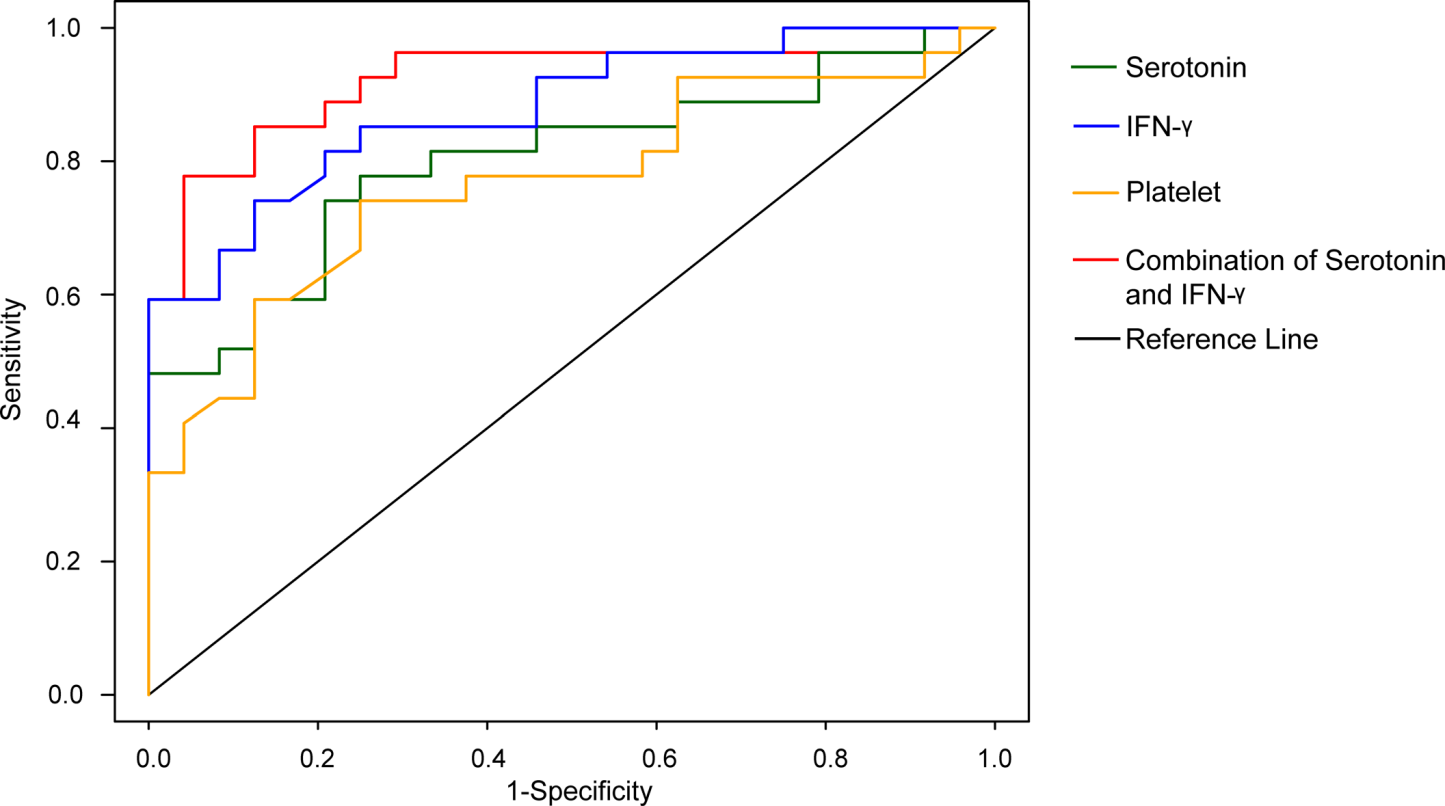
Receiving Operating Curve of combined serum serotonin and IFN-γ. Values indicate prediction performance for DHF. Green, serotonin only; blue, IFN-γ only; yellow, platelets only and red, serotonin and IFN-γ. The receiver operating characteristic (ROC) curve plot is a plot of the true positive rate (sensitivity) against the false positive rate (1—specificity) for the different possible cutpoints of a prognostic test, and provides a useful metric to compare different indicator variables (biomarkers). An area under the ROC curve (AUC) value close to 1 indicates an excellent prognostic test, a curve that lies close to the diagonal (AUC = 0.5) has no information content and therefore no prognostic utility. Amongst various sera metabolites and cytokines, the highest AUC value (0.92) was the combination of serotonin and IFN-γ.

To improve on serotonin predictive ability and extend the scope of capturing inflammatory compounds in dengue infections as prognostic biomarkers of DHF, we performed multiplex immunoassays on 27 cytokines/chemokines. Nine were significantly different between DF and DHF namely, IFN-γ, IL-1β, IL-4, IL-8, G-CSF, MIP-1β, FGF basic, TNFα and RANTES (S7 Fig). VIP plots generated from OPLS-DA modelling revealed IFN-γ as the top ranked cytokine in dengue infections (S8A Fig). The importance of IFN-γ as a prognostic biomarker is consistent with it being widely reported as an important pro-inflammatory cytokine in dengue [22,23]. In addition, due to reports of the elevation of IL-10 in DHF patients, IL-10 has been suggested as a biomarker of severe dengue [24,25]. We compared the DHF prediction potential for IFN-γ alone, IL-10 alone, platelets alone, and the combination of serotonin and IFN-γ at <96 h from onset of fever. The AUCs of platelets, serotonin alone, IFN-γ alone and IL-10 alone were 0.78 (95% C.I. 0.66–0.90, *p* = 0.0001), 0.80 (95% C.I. 0.68–0.92, *p* = 0.0002), 0.88 (95% C.I. 0.79–0.97, *p*<0.0001), and 0.55 (95% C.I. 0.40–0.69), respectively (S8B and S8C Fig). We combined serotonin and IFN-γ which resulted in AUC of 0.92, sensitivity = 77.8% and specificity = 95.8% (*p*<0.0001; Fig 5) in predicting DHF.

## Discussion

Through a combined untargeted and targeted metabolomics screen in dengue patients, we have identified depressed levels of circulating serotonin in DF and DHF patients. Lower serotonin levels in DF patients have been observed previously and are consistent with our study [26]. Our study thus extended on previous knowledge by demonstrating that the declines of serum serotonin are steeper in DHF patients compared to DF patients in the febrile phase and this declining trend continues into the defervescence phase. Due to early drop in circulating serotonin within the first 96 h from onset of fever, we propose that serotonin, and the inclusion of cytokines, such as IFN-γ may be used as prognostic biomarkers for the early prognosis of DHF.

Circulating serotonin forms a distinct pool separate from central nervous system serotonin pool, and is taken up via SERT and stored in platelets. During platelet aggregation, platelet-stored serotonin is released into circulation [27], which in turn promotes platelet aggregation in a feedback fashion via the serotonin receptor (5-HT_2A_) on platelets [28]. It has been shown that sera from DHF patients cross-react with platelets and inhibit platelet aggregation due to the auto-antibodies directed against DENV nonstructural protein 1 (NS1) [29,30]. Anti-NS1 antibodies bind to platelet membrane protein disulfide isomerase (PDI) [31]. Moreover, these anti-NS1 autoantibodies induce platelet lysis [30]. Given that systemic NS1 protein levels correlate with viremia and dengue severity [32,33], it is conceivable that attenuated platelet aggregation and numbers in dengue patients may have resulted in the depressed serum serotonin levels and even more so in DHF patients. In addition, animal models and human *ex vivo* studies have shown that NS1 cross-reacts with platelets and endothelial cells and reduce platelet aggregation through the generation of auto-anti-platelet antibodies [30,31,34]. Notably, similar levels of HTP, the precursor and HIAA, the main metabolite of serotonin, were found in DF and DHF, suggesting aberrations in the release and/or uptake of serotonin rather than alterations in serotonin metabolism. The targeting of SERT by serotonin-selective reuptake inhibitors (SSRIs) which inhibit the uptake and storage of platelet-serotonin is known to decrease platelet aggregation responses and consequently increase bleeding time [35]. Indeed, plasma leakage is the defining feature of severe dengue [14] and platelets are critical in maintaining the integrity of the vascular system. The steeper declines in serum serotonin in DHF patients in both febrile and defervescence phases suggest why plasma leakage occurs in DHF patients rather than DF patients. Therefore, our study provides mechanistic clues to how thrombocytopenia, steep serotonin decrease and plasma leakage may be linked in severe dengue.

In dengue, the mechanisms leading to thrombocytopenia are poorly understood and could occur via several modes, including peripheral platelet destruction by the host immune system [6,36–38], bone marrow aplasia [39], aberrant platelet function or signaling [6], or all of the above. Recent studies reveal that platelets participate in inflammation by influencing adaptive immunity through interactions with monocytes, neutrophils and endothelial cells [8,40]. In addition several studies have suggested its role in maintaining patent capillary barrier. In experimental models, platelets adhere to endothelial cells infected with DENV2 [41], and directly interact with monocytes and neutrophils [42]. Neutrophils recruited to injured vessels under pathogenic inflammatory conditions selectively capture activated platelets [43]. In humans, platelets were reported to form platelet-leukocyte and platelet-monocyte aggregates [44]. Serotonin is cross-linked to a variety of platelet surface adhesion proteins and clotting factors [45,46] required for platelet aggregation and interaction with other cell types. It is not clear, however, how diminished serotonin in DF and DHF patients affects these platelet-cell interactions.

Given the more rapid decline in serum serotonin in DHF patients compared to DF patients as early as 96 h after fever onset, we propose serum serotonin as a predictive marker of severe dengue prognosis. We and others previously identified IFN-γ as a candidate early prognosis biomarker [22,47] and we integrated serotonin and IFN-γ to achieve early prediction of patients likely to develop DHF at sensitivity of 77.8% and specificity of 95.8% within 96 h of fever onset. Our proposed serotonin with IFN-γ duplex biomarker panel attained the same prognostic performance of 0.92 compared to an eight feature panel previously identified [47]. This may simplify prognosis in a clinical setting. Serotonin and IFN-γ reflects the pathobiology of dengue-mediated thrombocytopenia and systemic inflammation respectively, and this information makes these candidate biomarkers biologically significant and plausible in their reflection of the pathognomonic symptoms of severe dengue. In this study, we show that serotonin levels in DHF declined more than DF patients in the febrile phase and continued to stay suppressed in the defervescence phase. Using circulating serotonin as a dengue prognosis biomarker appears to have its benefits–we demonstrated that its levels and association with thrombocytopenia in dengue is independent of whether the infection is primary or secondary. Future technological developments into rapid and cheaper analytical methods of serotonin and IFN-γ levels may facilitate early prognosis even in dengue-endemic, resource-poor areas lacking laboratory facilities, although the predictive performance of serotonin and IFN-γ needs further validation in a separate cohort. Measuring serum serotonin levels in other febrile, acute infectious disease, as well as in patients infected with different DENV type may aid in further showing specificity and potential universality of serotonin as a reliable biomarker.

## Supporting Information

S1 Fig Experimental design and workflow of the study. Subjects were recruited into the study and their blood collected over the course of study. Untargeted metabolomics was performed on 25 DF and 27 DHF patients only at the febrile phase. Next, targeted metabolomics was performed on patients from all three phases: febrile stage (25 DF, 27 DHF), defervescence stage (31 DF, 29 DHF), and convalescence stage (25 DF, 25 DHF).
(PDF)

S2 Fig Chromatogram of serotonin,5-hydroxy-indole-3-acetic acid and 5-hydroxytryptophan. The analytes are separated by reverse-phase chromatography, and quantification via the use of spiked, known concentrations of deuterated internal standards serotonin-d4 for serotonin, 5-hydroxy-indole-3-acetic acid and 5-hydroxytryptophan, and kynurenine-d4 for kynurenine. The ratios of the endogenous metabolite to their respective internal standards are then intrapolated to their corresponding standard curves to determine their concentrations
(PDF)

S3 Fig Serotonin levels in primary (open) and secondary (brown) dengue patients. (PDF)

S4 Fig Relationship of serum serotonin and platelets in dengue. (A and B) Correlation of platelet numbers with serotonin levels in the febrile and defervesence phases in DF and DHF patients. (C and D) Serotonin concentration and platelet numbers in DF and DHF as a function of time (day).
(PDF)

S5 Fig Temporal profile of circulating 5-hydroxy-indole-3-acetic acid (HIAA) and 5-hydroxytryptophan (HTP) in the three phases of dengue. Temporal profiles of (A) HTP and (B) HIAA across the febrile (1–4 d), defervescence (5–7 d) and convalescence (21–28 d) phases. Mean value (middle lines) ± standard deviation (SD, error bars) is reported. Significance is indicated as *, *p*<0.05; **, *p*<0.01; ***, *p*<0.001 by Mann Whitney test.
(PDF)

S6 Fig **Receiver Operating Curves of serotonin (A) and kynurenine (B).** The sensitivity and specificity refer to distinguishing DF and DHF.
(PDF)

S7 Fig Differential serum cytokines in DF and DHF. The cytokine data represent the febrile phase of the infection.
(PDF)

S8 Fig Feature evaluation and selection for optimal severe dengue prognostication. (A) Top ranking cytokines and chemokines generated from VIP scoring. (B and C) Receiver Operating Curves of platelets (B) and IFN-γ (C). The sensitivity and specificity refer to distinguishing DF and DHF.
(PDF)

S1 Table Differential metabolites at levels 3 and 4 based on Metabolomics Standard Initiative. (XLSX)
