# Supplementary figures and images for: Serum Metabolomics Reveals Serotonin as a Predictor of Severe Dengue in the Early Phase of Dengue Fever
Source: PLoS Negl Trop Dis. 2016 Apr 7;10(4):e0004607. doi: 10.1371/journal.pntd.0004607 (PMC4824427; doi:10.1371/journal.pntd.0004607)

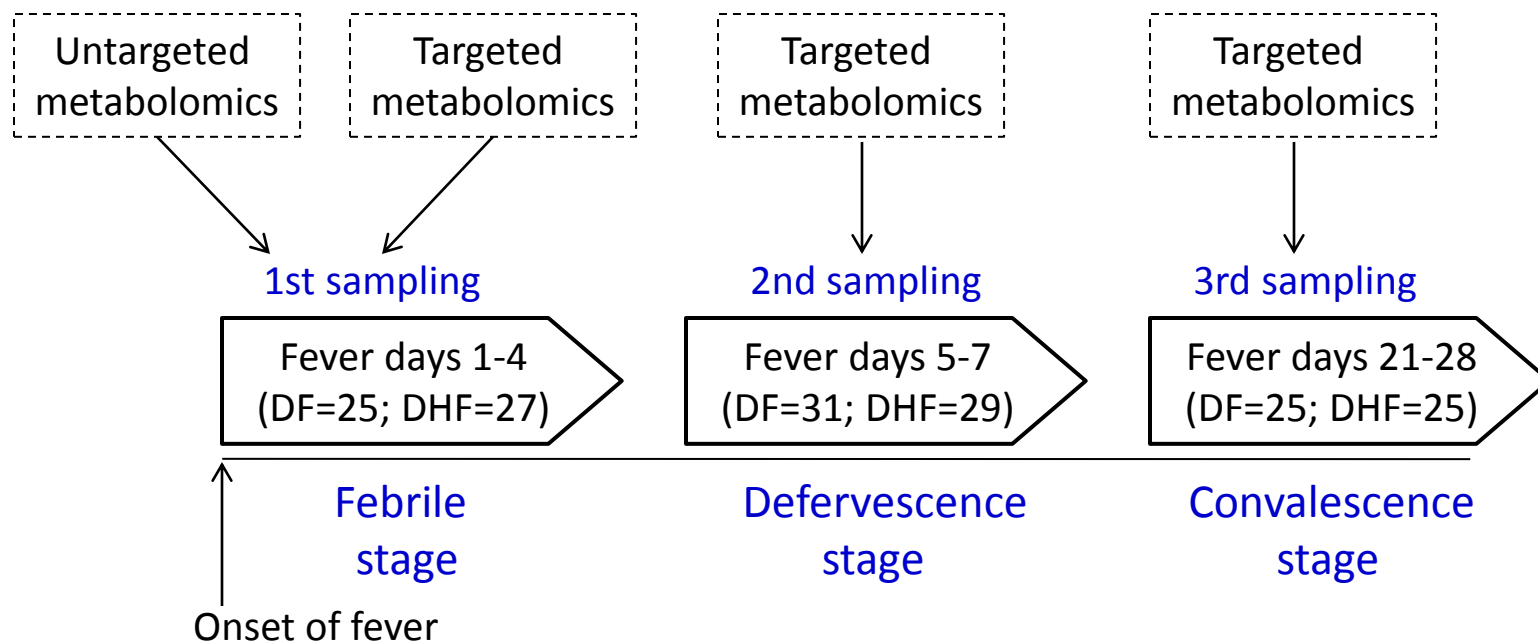

Supplement: S1 Fig — Subjects were recruited into the study and their blood collected over the course of study. Untargeted metabolomics was performed on 25 DF and 27 DHF patients only at the febrile phase. Next, targeted metabolomics was performed on patients from all three phases: febrile stage (25 DF, 27 DHF), defervescence stage (31 DF, 29 DHF), and convalescence stage (25 DF, 25 DHF). (PDF) [file pntd.0004607.s001.pdf]

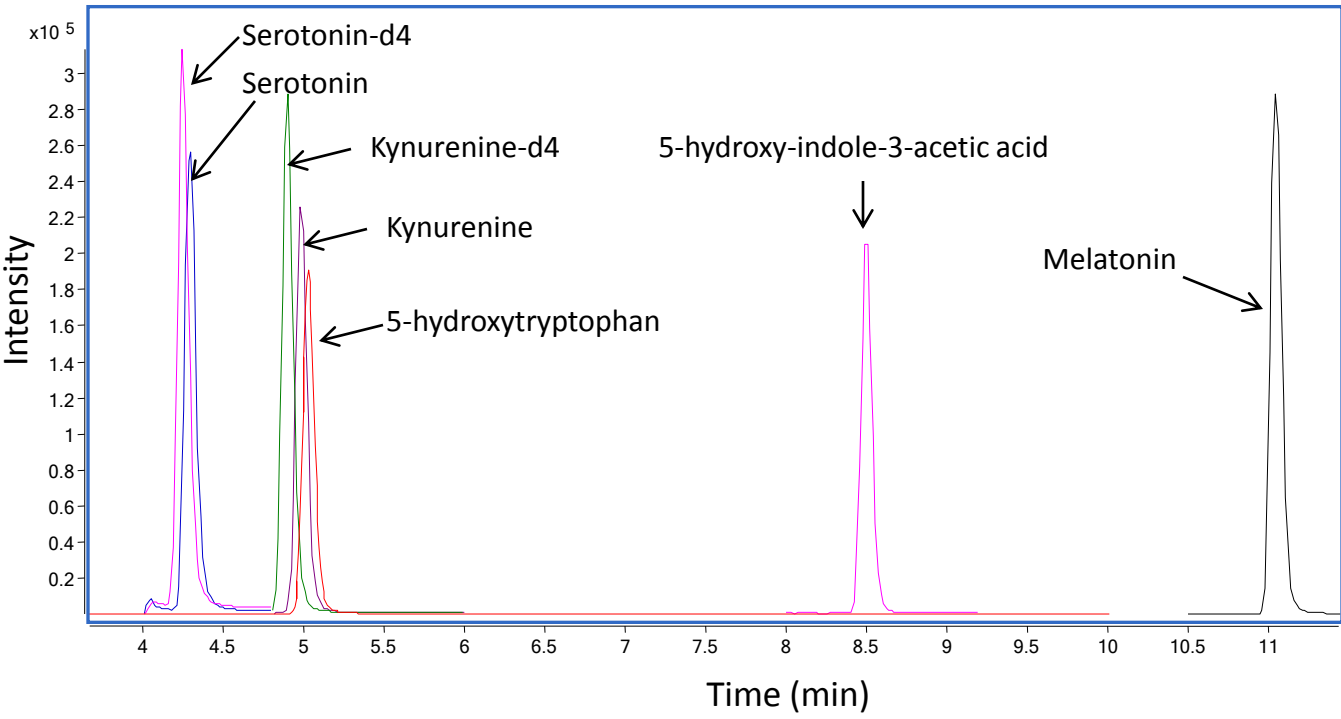

Supplement: S2 Fig — The analytes are separated by reverse-phase chromatography, and quantification via the use of spiked, known concentrations of deuterated internal standards serotonin-d4 for serotonin, 5-hydroxy-indole-3-acetic acid and 5-hydroxytryptophan, and kynurenine-d4 for kynurenine. The ratios of the endogenous metabolite to their respective internal standards are then intrapolated to their corresponding standard curves to determine their concentrations (PDF) [file pntd.0004607.s002.pdf]

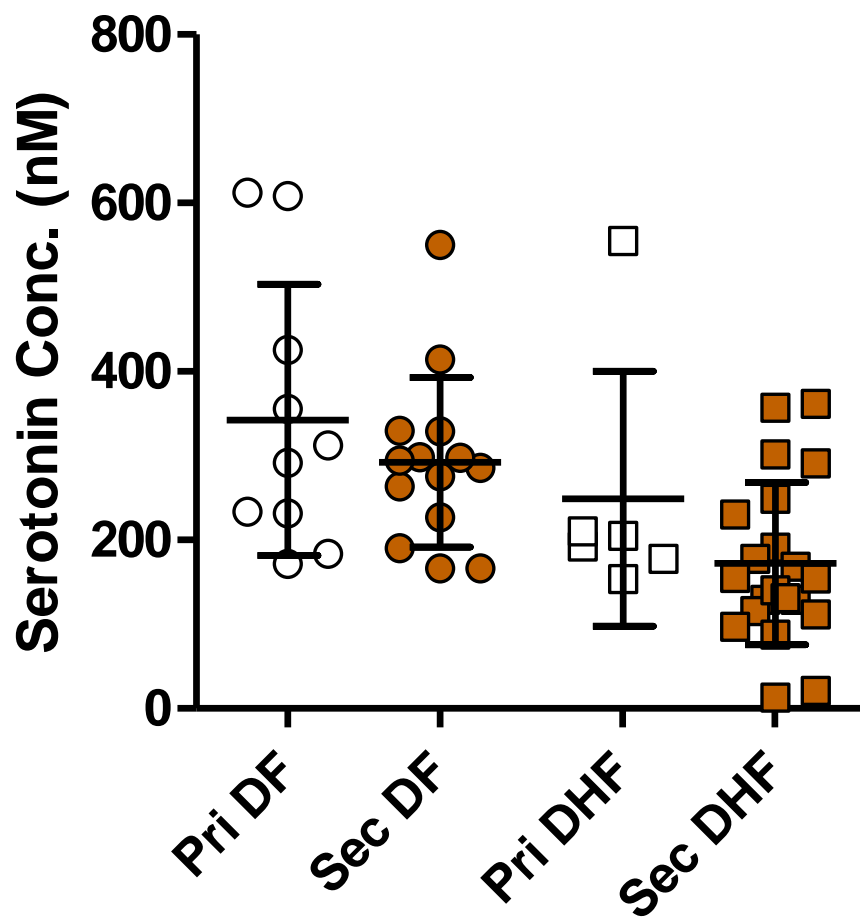

Supplement: S3 Fig — (PDF) [file pntd.0004607.s003.pdf]

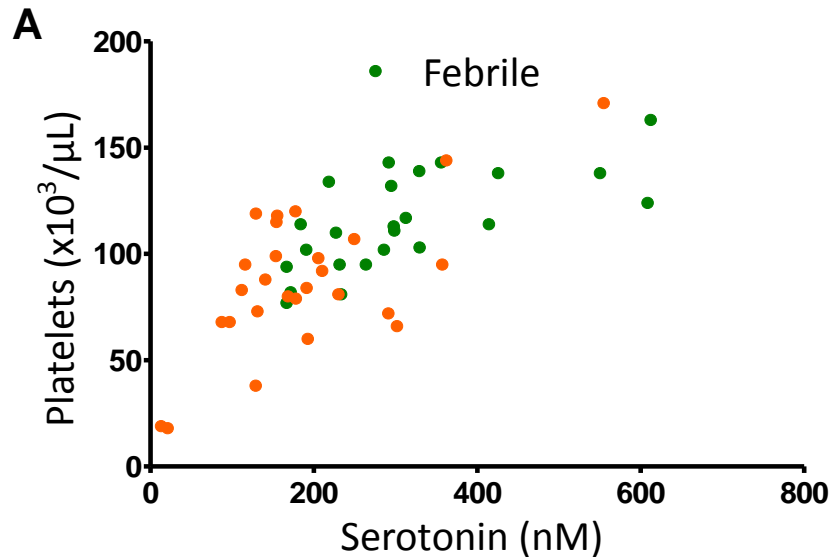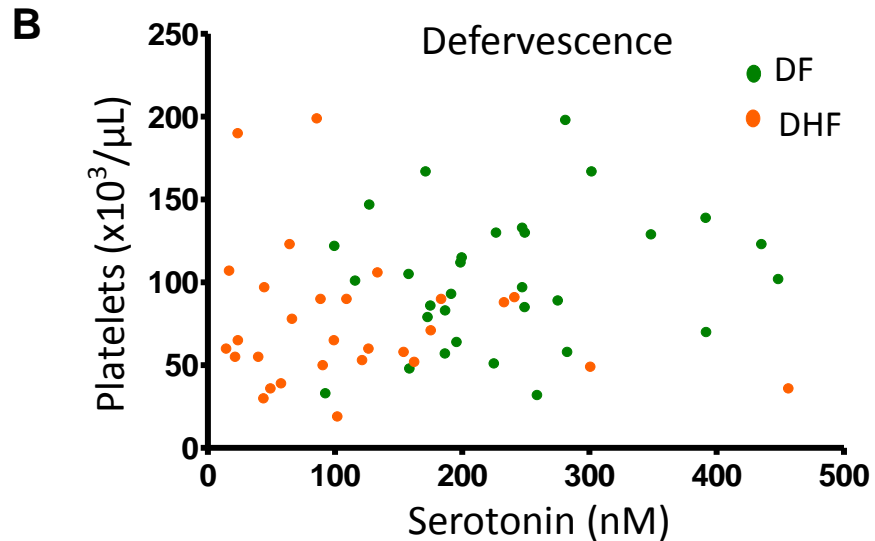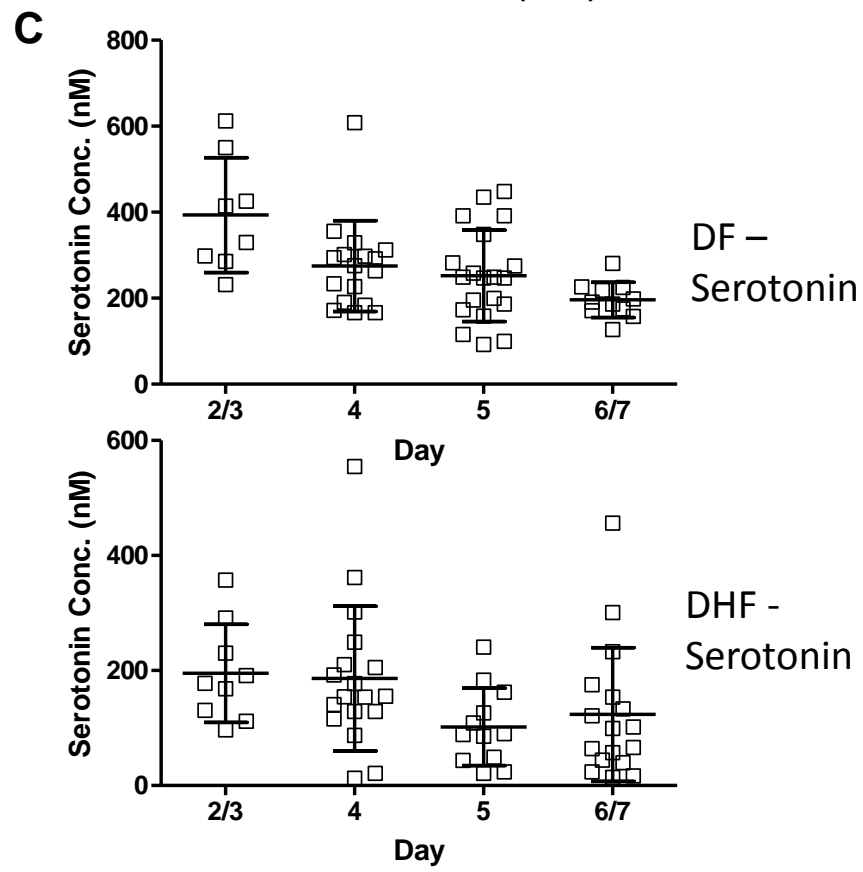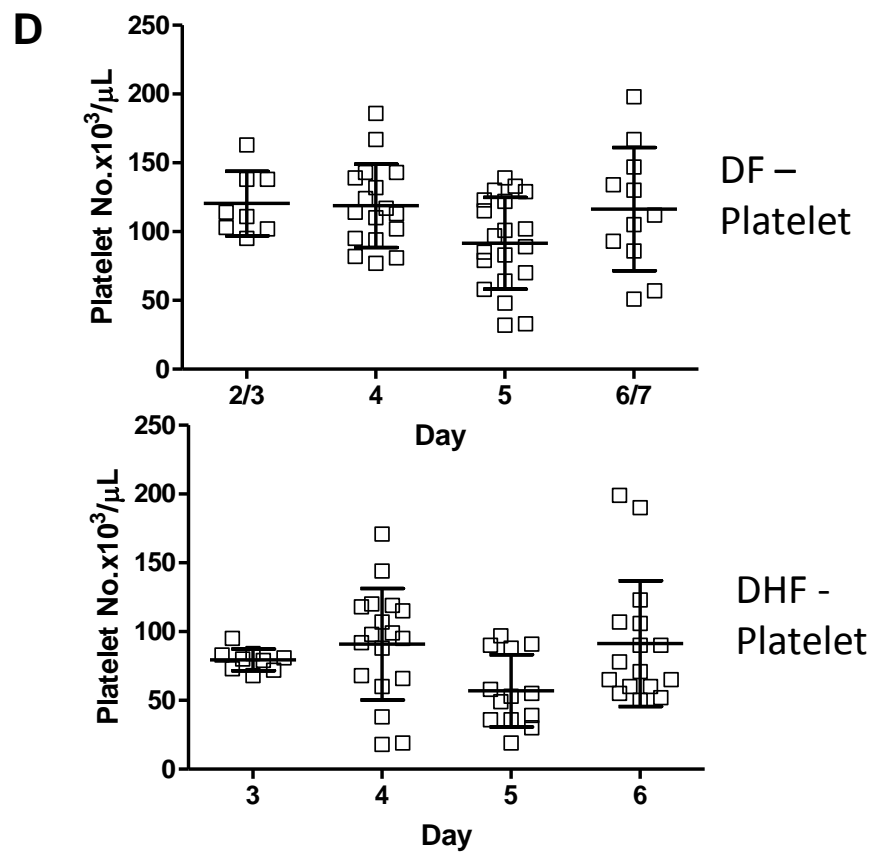

Supplement: S4 Fig — (A and B) Correlation of platelet numbers with serotonin levels in the febrile and defervesence phases in DF and DHF patients. (C and D) Serotonin concentration and platelet numbers in DF and DHF as a function of time (day). (PDF) [file pntd.0004607.s004.pdf]

**A**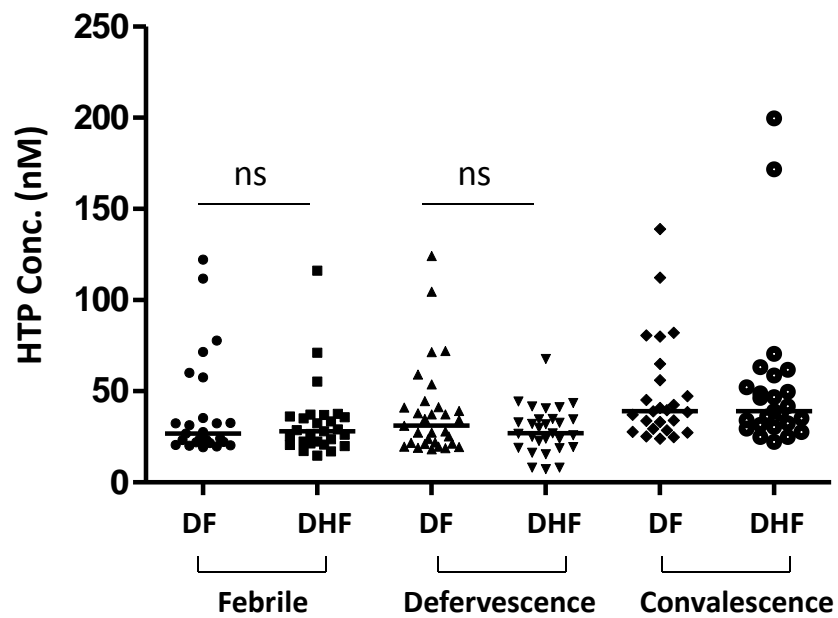**B**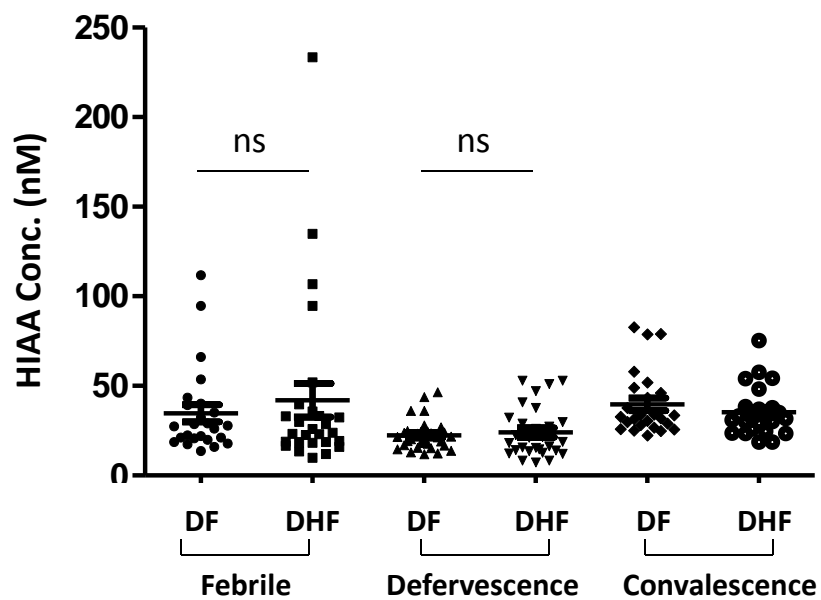

Supplement: S5 Fig — Temporal profiles of (A) HTP and (B) HIAA across the febrile (1–4 d), defervescence (5–7 d) and convalescence (21–28 d) phases. Mean value (middle lines) ± standard deviation (SD, error bars) is reported. Significance is indicated as *, p<0.05; **, p<0.01; ***, p<0.001 by Mann Whitney test. (PDF) [file pntd.0004607.s005.pdf]

IFN $\gamma$ 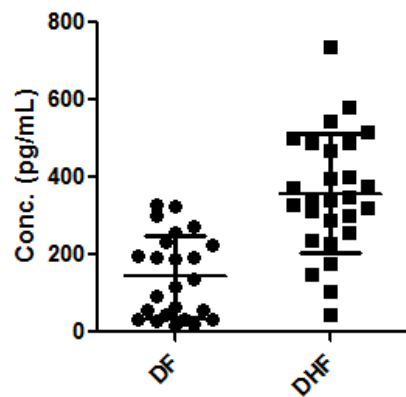

IL1b

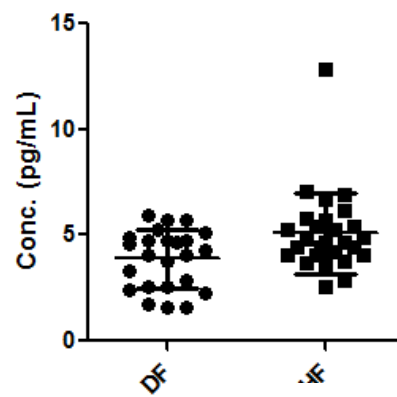

IL4

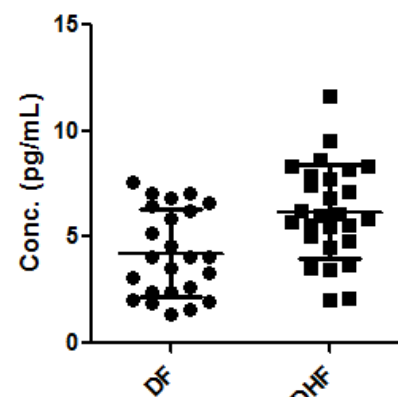

IL8

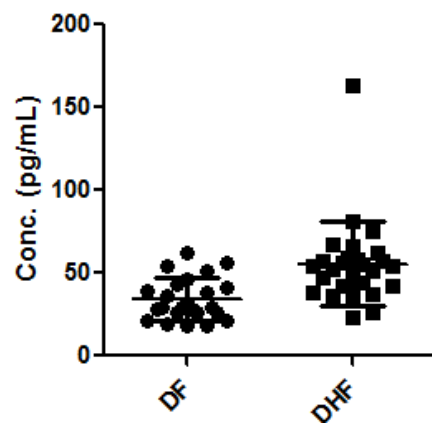

MIP1b

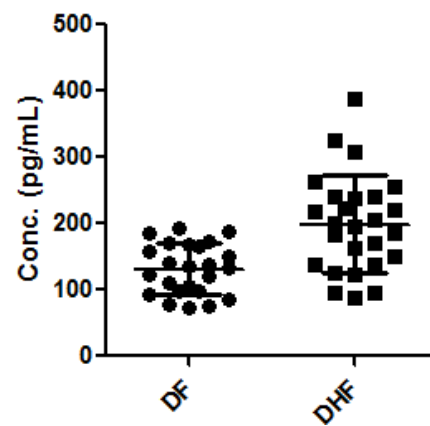

FGFbasic

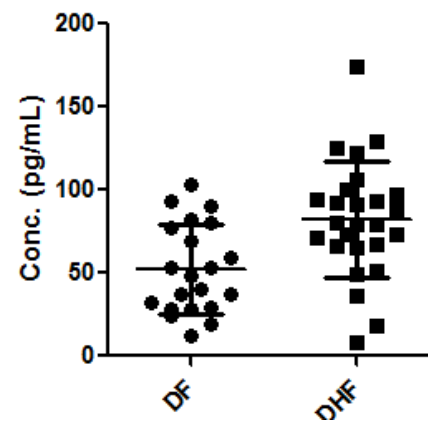

IL-10

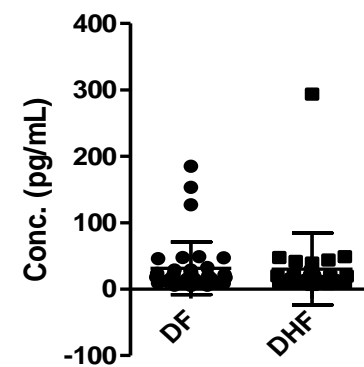

G-CSF

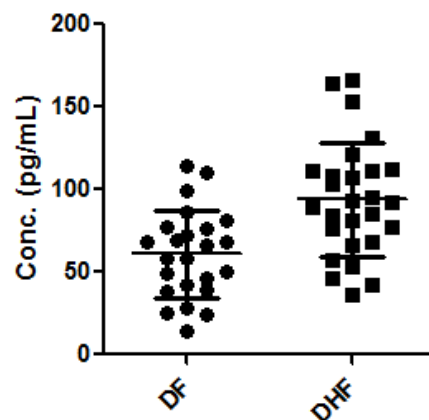TNF $\alpha$ 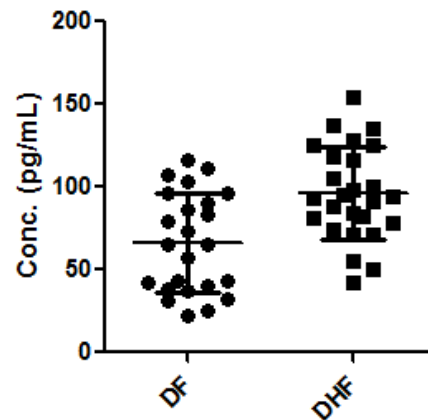

RANTES

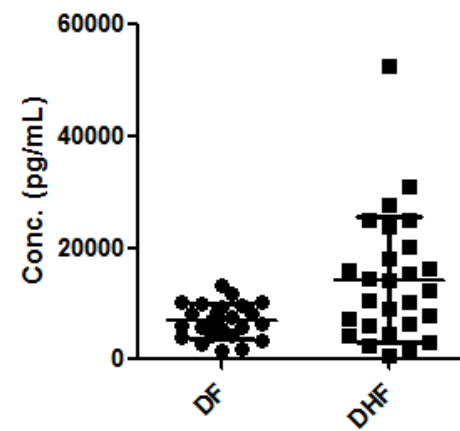

Supplement: S7 Fig — The cytokine data represent the febrile phase of the infection. (PDF) [file pntd.0004607.s007.pdf]

**A**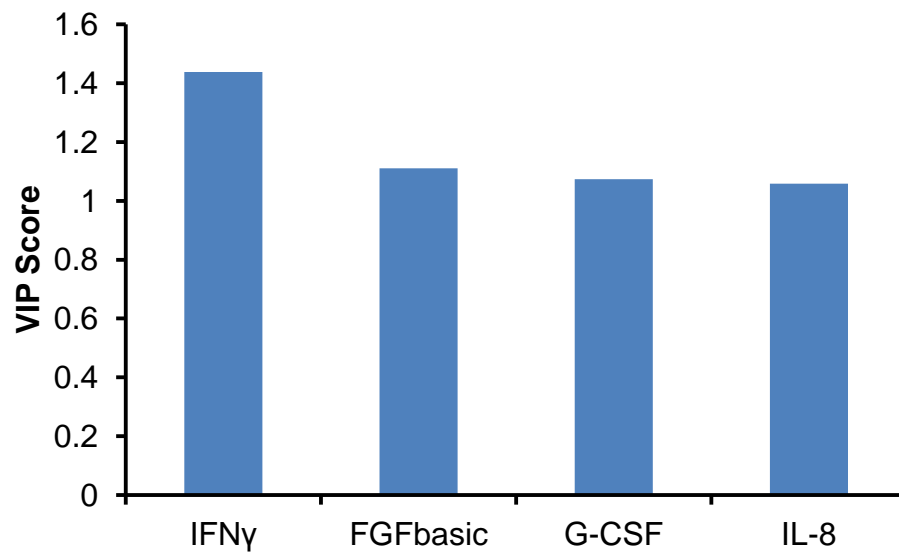**B**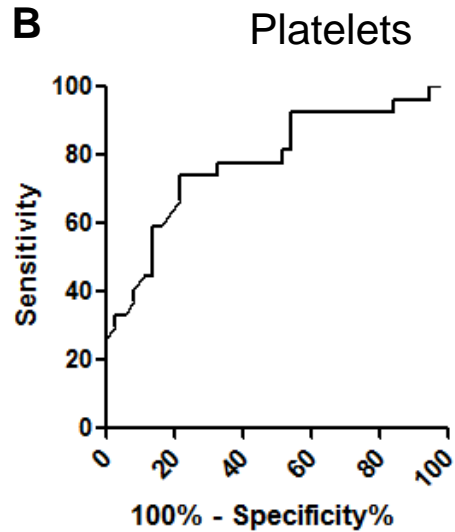

|                         |              |
|-------------------------|--------------|
| Area                    | 0.78         |
| Std. Error              | 0.06         |
| 95% confidence interval | 0.66 to 0.90 |
| P value                 | 0.0001       |

**C**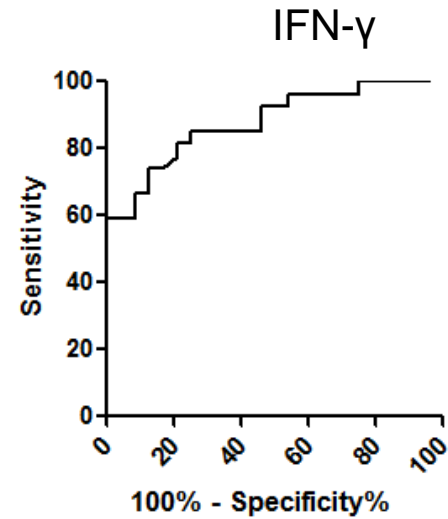

|                         |              |
|-------------------------|--------------|
| Area                    | 0.88         |
| Std. Error              | 0.05         |
| 95% confidence interval | 0.79 to 0.97 |
| P value                 | < 0.0001     |

Supplement: S8 Fig — (A) Top ranking cytokines and chemokines generated from VIP scoring. (B and C) Receiver Operating Curves of platelets (B) and IFN-γ (C). The sensitivity and specificity refer to distinguishing DF and DHF. (PDF) [file pntd.0004607.s008.pdf]
