# Supplementary material for: Serum Metabolomics Reveals Serotonin as a Predictor of Severe Dengue in the Early Phase of Dengue Fever
Source: PLoS Negl Trop Dis. 2016 Apr 7;10(4):e0004607. doi: 10.1371/journal.pntd.0004607 (PMC4824427; doi:10.1371/journal.pntd.0004607)
Supplement: S6 Fig — Receiver Operating Curves of serotonin (A) and kynurenine (B). The sensitivity and specificity refer to distinguishing DF and DHF. (PDF) [file pntd.0004607.s006.pdf]

A

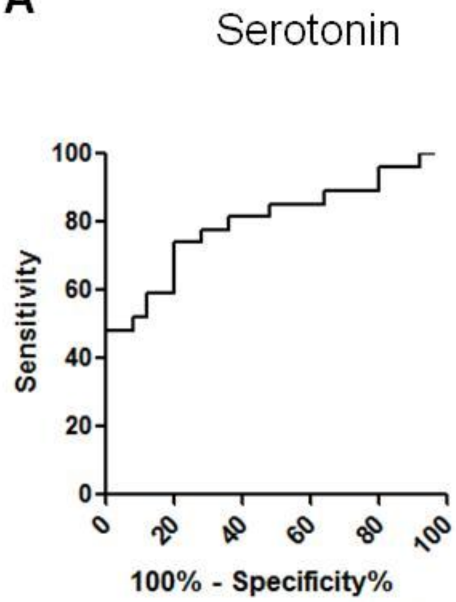

Area	0.80
Std. Error	0.06
95% confidence interval	0.68 to 0.92
P value	0.0002

B

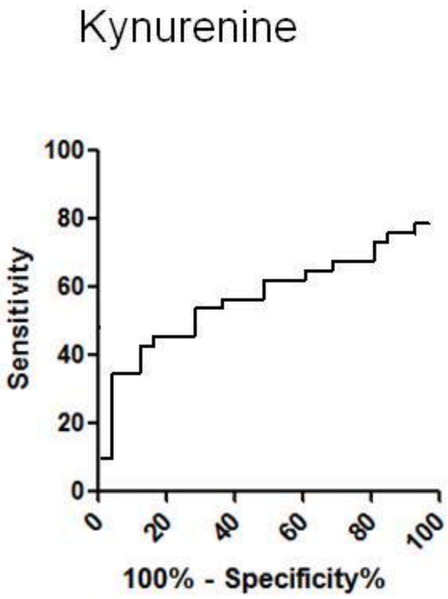

Area	0.72
Std. Error	0.07
95% confidence interval	0.57 to 0.85
P value	0.008
